# Hydrodeoxygenation of Xylose Isopropylidene Ketal Over Pd/HBEA Catalyst for the Production of Green Fuels

**DOI:** 10.3389/fchem.2021.729787

**Published:** 2021-08-20

**Authors:** Matheus O. Souza, Sergio C. Pereira, Lam Y. Lau, Leandro Soter, Marcelo M. Pereira

**Affiliations:** ^1^ Instituto de Química, Universidade Federal do Rio de Janeiro, Rio de Janeiro, Brazil; ^2^ Instituto de Química, Universidade do Estado do Rio de Janeiro, Rio de Janeiro, Brazil

**Keywords:** hydrodeoxygenation, Pd/HBEA catalyst, xylose isopropylidene ketal, hydrocarbons, green fuels

## Abstract

1,2:3,5-Di-O-isopropylidene-α-D-xylofuranose (DX) is a major component of a new bio-crude: a viscous oil presenting petroleum-friendly properties produced by the ketalization of sugarcane bagasse. This article studies DX HDO (hydrodeoxygenation) over a Pd/HBEA catalyst in a batch reactor at 250°C*.* The effects of hydrogen pressure from 10 to 40 bar, catalyst/DX ratio from ½ to 2, and reaction time 0–24 h were investigated. A range of conditions for complete hydrodeoxygenated DX into alkanes with a Pd/HBEA catalyst was found. In these conditions, a low coke yield with water as the principal deoxygenated product was obtained. Further, higher amounts of alkanes containing seven or more carbons (A_7+_) were favored at 30 bar of hydrogen pressure, Cat/DX ratio = 2, and short reaction time. Products analysis that accompanied the above variations during reaction time led to general insights into reaction pathways. First, in the presence of DX, an effective n-hexane conversion was not observed on experiments of low catalyst/DX ratio (½) or in the initial period of high Cat/DX ratio, suggesting DX is much more successful than n-hexane to compete for active sites*.* Then, the formation of a pool of oxygenated compounds, such as furans, ketones, and carboxylic acids, along with lighter and heavier alkanes was observed. Hence, the aforementioned oxygenates may undergo reactions, such as aldol condensation with subsequent hydrodeoxygenation reaction, generating heavier alkanes.

## Introduction

In the last decades, the increase of greenhouse gas (GHG) emissions has induced environmental awareness, which led to developing policies concerning the implementation of clean energy sources worldwide (French Government, 2015). In this scenario, biomass could be considered the major feedstock capable of flattening the curve of GHG emissions in the short term due to its year-round availability and low cost (Saghaei et al., 2020).

The conversion of biomass into fuels could be done through gasification to syngas and subsequent hydrocarbon production through the Fischer–Tropsch process and liquefaction to produce ethanol through fermentation or bio-oil (Wyman, 1999; Demirbaş and Arin, 2002; Wang et al., 2021). Among them, pyrolysis gained importance over the last 2 decades. It could be described as a simple thermochemical process under an inert or a low oxygen content atmosphere at temperatures around 500°C and short residence times (Mettler et al., 2012). However, a considerable loss of mass occurs during the conversion of biomass into bio-oil. In addition, bio-oil is a complex mixture mainly composed of oxygenated molecules (e.g., phenols and acetic acid), which confers high acidity, corrosiveness, and instability (Lehto et al., 2014) (Xiu and Shahbazi, 2012) (Oasmaa et al., 2012). Thus, despite the simplicity of the pyrolysis process, several drawbacks should be overcome to achieve sequential bio-oil conversion in a typical oil refinery process.

As an alternative approach, our group developed two steps for the conversion of biomass into liquid fuel. First, sugarcane bagasse is converted into a viscous, chemically stable, dark-brown bio-petroleum (BP) oil through simultaneous hydrolysis-ketalization reactions. This transformation adds mass to BP and avoids parallel reactions. It is composed mainly of carbohydrate isopropylidene ketals, where 1,2:3,5-di-*O*-isopropylidene-α-D-xylofuranose (DX) is one of the major compounds (Garrett et al., 2015; dos Santos et al., 2020). Thus, DX can be used as a feed for further conversion or as a bio-crude representative compound. Previous works have demonstrated the DX conversion using fluidized catalytic cracking (FCC) and hydrodeoxygenation (HDO). The former produced mainly aromatics and light olefins. The latter produced aliphatic, cyclic, and aromatic hydrocarbons (Batalha et al., 2014, Batalha et al., 2017; Pinto et al., 2019a, Pinto et al., 2019b; Pereira et al., 2020, Pereira et al., 2021). Further, the wide variety of metal/support catalysts used in the HDO process is an advantage since it enables the conversion of different biomass-derived molecules into green fuels. Examples encompass the HDO of pyrolysis compounds such as phenol, anisole, and guaiacol over the noble metal/BEA catalyst (Zhu et al., 2011; Li et al., 2017) and lignin depolymerization into monoaromatics and bio-oil over Ni/C catalyst (Wang et al., 2019).

Our group explored this process’ advantages to both deoxygenated DX and produced long carbon-chain hydrocarbons. An initial screening of catalysts on a fixed bed reactor concluded that the catalysts based on Pd supported on zeolites, such as Pd/HBEA and Pd/HZSM-5, presented more selectivity to convert DX into hydrocarbons and less coke yield. From these initial results, Pd/HZSM-5 was selected and applied to DX HDO on a batch reactor. Furthermore, a mesoporous Pd/ZSM-5 was explored, which enhanced the hydrocarbons’ yield with seven or more carbons compared with normal HZSM-5. In recent works, two catalysts, Cu-Pd/HZSM-5 and Cu-Pd/HBEA [both containing 0.01% Cu(w/w) and 0.5% Pd (w/w)], were studied as a catalyst for HDO of 15% (w/w) DX in n-hexane for 24 h at 20°C under 40 bar of H_2_. Both catalysts converted DX into hydrocarbons containing up to ten carbon atoms, such as trimethyl-benzene, xylenes, branched, linear paraffin, and cycloalkanes. More importantly, Cu-Pd/HBEA catalyst yielded more cyclic hydrocarbons when compared with Cu-Pd-HZSM-5. DX has only 11 carbons; in contrast, large compounds (8–30 carbon-chain) are presented in BP. Thus, a further advantage of the mesoporous area is expected in converting large molecules.

Hence, these works suggested that HBEA with a larger pore opening and a higher external surface than ZSM-5 zeolites deserves further attention. This article aims to study DX HDO over Pd/HBEA catalyst. For this purpose, we use a batch reactor for evaluating several parameters. Moreover, we included a Pd/ZSM-5 for comparison. Our focus was to maximize the yield of cyclic hydrocarbons with six or more carbons (CyAlk_6+_) along with linear or branched hydrocarbons with seven or more carbons (Alk_7+_). Additionally, we presented insights into DX conversion into hydrocarbons.

## Experimental

### Catalysts Preparation

BEA (Si/Al ratio = 23; cod. CP-814E produced by Zeolyst International Inc., United States) and ZSM-5 (Si/Al ratio = 23; from CENPES, Petrobras Research Center, Brazil) were used. Prior to Pd impregnation, both zeolites were converted to their protonated forms by exchanging with 1 M (NH_4_)_2_SO_4_ solution, then washing with hot water (80°C), and being thermally treated at 450°C for 3 h; they were named HBEA and HZSM-5. Pd was deposited using incipient wetness as follows: 40 ml of Pd aqueous solution [0.44 g of Pd(NO_3_)_2_.5H_2_O dissolved into 40 ml H_2_O] was gently added dropwise and manually mixed with 35 g of zeolite. The resultant slurry was first dried at 130°C for 24 h and then calcinated at 450°C for 3 h.

### Catalyst Characterization

The metal content on the catalyst was determined by the Supermini200 X-ray fluorescence spectrometer (Rigaku). Detection was performed by PC and SC detectors with an element detection range from F to U. X-ray source was constituted by a Pd lamp set on 50 kV and 4.0 mA.

Determination of catalyst textural properties was carried out on the Quantachrome Nova e-4200 physisorption. First, the catalyst was pre-treated by heating from room temperature to 220°C; then, the temperature was maintained at 220°C under vacuum for 16 h. After the pre-treatment, adsorption-desorption isotherms were determined at 77 K. The specific surface area (S_BET_) and external specific area (S_ext_) were calculated using Brunauer–Emmett–Teller (BET) method and t-plot, respectively. Total pore volume was determined from BJH fitting, and micropore volume was determined from the t-plot.

Catalyst X-ray spectrum was carried out on the Ultima IV diffractometer produced by Rigaku Corporation. The radiation source consisted of Cu K-α1 source equipped with a Ni filter (λ = 0,15,406 ηm). Spectra were collected in the 2θ angle range 5°–80° by an angle increment of 0.02° during 0.16 s each.

Brønsted and Lewis sites were measured by pyridine adsorption-desorption monitored by the FTIR spectrometer model IRPrestige-21 produced by Shimadzu Inc. 0.1 g of calcined catalyst and 1 ml of pyridine were inserted into a vial. Then, the vessel was closed and shaken manually for 24 h. Then, pyridine in excess was evaporated (at 100°C for 3 h). Dry samples were then analyzed on a FTIR spectrometer with a wavenumber range from 1,350 to 1,650 cm^−1^ and a resolution equal to 2 cm^−1^, and 16 scans were carried out. Background spectra were obtained from calcined samples without pyridine. The modification on acid sites by metal was estimated based on a relative quantitation between the peak heights of some specific peaks, such as the Lewis acid site (1,446 cm^−1^) and Brønsted acid site (1,542 cm^−1^).

### DX Synthesis

A 30 g of D-xylose was suspended in 800 ml of acetone and cooled to 10°C in an ice bath. Then, 20 ml of H_2_SO_4_ (98% purity) was added dropwise to the cooled suspension for 15 min and the system was heated to 20°C and kept at this temperature under magnetic stirring for 90 min. The resultant light-yellow solution was cooled to 10°C and neutralized through dropwise addition of an 80 ml NaOH (40% wt.) aqueous solution. The resultant suspension was filtered under vacuum, and the filtrate was introduced into the rotary evaporator in order to evaporate acetone under low pressure at 35°C. From this step, a white emulsion residue was obtained, which was mixed with 120 ml of ethyl acetate, forming two phases. The aqueous phase was separated, and the organic phase was washed two times with 15 ml H_2_O and inserted into a rotary evaporator to evaporate ethyl acetate under low pressure at 35°C. The residual transparent oil mainly consisted of DX and was washed with n-hexane to extract DX. Afterward, n-hexane was evaporated under reduced pressure, and DX was isolated (65% yield) as presented elsewhere (Garrett et al., 2015).

### Catalytic Tests

Catalytic tests were carried out in a 450 ml stainless steel autoclave reactor, model Parr 4848. Feed compositions, temperature, hydrogen pressure, and reaction times were the variables explored. Prior to HDO reactions, Pd was reduced and water in zeolite support was minimized *in situ* in a one-step process as follows: the catalyst was left inside the reactor under a hydrogen flow of 120 ml/min for 3 h at 285°C, 3 bar, and 300 RPM. Afterward, the reactor’s temperature was lowered to room temperature; the internal pressure was equalized to the atmospheric pressure. Then, pure n-hexane or mixture with DX was introduced into the reactor through a syringe plugged on the reactor’s head.

Then, the hydrogen pressure and stirring were adjusted and the reactor was heated (at a rate of 15 °C/min) to the desired temperature. Reactions were finalized by immersing the reactor vessel in an ice bath. Some tests were performed in duplicate, and error in conversion was less than 5% and selectivity less than 10%. After a group of nine reactions, we repeated a test and observed no considerable changes in conversion and selectivity between tests. Thus, after each reaction, the catalyst was regenerated using the same calcination conditions (previously presented) and reused up to ten reactions.

### Liquid Products Analysis

Liquid products were weighed on an analytical balance. Liquid product analysis was split into two parts: 1) identification and quantitation (qualitative and quantitative analyses) of compounds produced by DX hydroconversion; 2) quantitation of n-hexane conversion. Each analysis was performed based upon an external standard calibration whose details are explained in the Supplementary Material.

In experiments wherein only products in the liquid phase were quantified, relative concentration was adopted as the method of comparison. Then, compound concentrations were determined using their absolute responses corrected by their respective response factors calculated from the calibration curves.

Identification and quantification in liquid products analysis were performed on a gas chromatograph (GC) model 7890A coupled to a flame ionization detector (FID) and a mass spectrometer (MS) model 5975C GC/MSD containing a quadrupole and a triple-axis detector. Samples were injected using an automatic sample injector model 7963A. The whole instrument was purchased from Agilent Technologies Inc. (United States).

A capillary column model HP1-MS (60 m × 250 μm x 0.25 μm; 100% dimethylpolysiloxane) was used under a constant flow of He equal to 0.8 ml/min. The oven temperature program was set as follows: 1) heating from 30°C to 120°C at a rate of 3°C/min; 2) heating from 120°C to 250°C at a rate of 10°C/min; 3) heating at 250°C for 5 min. FID was maintained under a hydrogen flow at 30 ml/min, an air flow at 400 ml/min, and a nitrogen makeup gas flow at 25 ml/min at 300°C. MS detector was operated on an electron impact ionization mode at an energy equal to 70 eV. The MS source was maintained at 230°C, while the quadrupole was maintained at 150°C. The MSD operated on a total ion monitoring setup with a mass range of 45–400 Da. The sample was injected into an injection chamber maintained at 250°C under a He flow equivalent to 0.8 ml/min. For the qualitative and quantitative analysis of DX hydroconversion compounds, the 1.0 μL sample was injected on a split ratio equal to 1:30, while for n-hexane conversion, the 0.1 μL sample was injected on a split ratio equal to 1:200.

The aqueous phase on liquid products was not observed. Hence, we suppose that zeolite catalyst absorbed the aqueous phase produced during hydroconversion reactions; therefore, the aqueous phase content was estimated from the oxygen balance. Equations used to calculate compounds or a class of compounds yields are described in detail in the Supplementary Material.

We classified the mono-oxygenated products containing oxygen into two types of intermediates. First, DX derivatives are ketal carbohydrates containing four and five oxygen such as 1,2-*O*-isopropylidene-α-D-xylofuranose and 1,6-anhydro-3,4-*O*-isopropylidene-2-*O*-methyl-β-d-galactopyranose. Oxygenates are mainly compounds like ketones, furfurals, and alcohols. Major compounds of oxygenates and DX derivatives are described in Supplementary Table 1.

### Gas Products Analysis

Gas mass was measured indirectly through brine mass shift. Aliquots of gas were transferred to a Büchner flask (BF) loaded with brine; then, they were confined into this apparatus at 298 K and 1 atm for further analysis. In this procedure, brine shifted by reactor gas was collected on a vessel and then weighted. The volume of gas is then calculated by the quotient of brine mass and its density. Using the value of gas volume, we could calculate, using the ideal gas equation, partial pressures of gaseous products and, therefore, their respective masses. Equations and apparatus used are described in detail in the Supplementary Material.

Each aliquot confined inside BF was injected (in triplicate) using a syringe into a micro gas chromatograph model, MicroGC 490, purchased from Agilent Technologies (United States). Gas products were performed in three different channels, and detection was performed using a thermal conductivity detector. Channels specifications were as follows: Channel 1 = Molsieve 5Å (10 m) maintained at 80°C under 150 kPa of Ar; Channel 2 = PoraPlot (10 m) maintained at 80°C under 150 kPa of He; Channel 3 = Al_2_O_3_/KCl (10 m) maintained at 100°C under 90 kPa of He. The injector was maintained at 110°C.

Retention times (RT) and molar response factors (MRF) were obtained using standard compounds 10% mol/mol in He. Equations and further information used to quantitate gas products are described in detail in the Supplementary Material.

### Coke Analysis

Determination of coke loading in the spent catalyst was performed using a thermogravimetric balance (TG). The instrument used was the TG model F1 209 Iris purchased from Netzsch GmBH (Germany). For analysis, a 10 mg of the sample (in an alumina crucible) was inserted into TG and heated as follows: 1) 35°C–250°C at a rate of 10°C/min under an N_2_ flow of 20 ml/min; 2) 250°C for 30 min under an N_2_ flow of 20 ml/min; 3) 250°C–700°C at a rate of 10°C/min under a synthetic air flow of 20 ml/min; 4) 700°C for 30 min under a synthetic air flow of 20 ml/min. TG balance was kept under an N_2_ purge flow of 10 ml/min during analysis. Content of coke loading (w/w) in the spent catalyst (%coke_catalyst_) was directly obtained from mass loss developed in the third heating rate program.

Mass of coke loading (m_coke_) was calculated based the following:

$$m_{\mathrm{coke}}\left(g\right)=m_{\mathrm{catalyst}}\times \%{\mathrm{coke}}_{\mathrm{catalyst}}/\left(1–\%{\mathrm{coke}}_{\mathrm{catalyst}}\right),$$ (1)

where m_catalyst_ is the mass of the loaded catalyst and %coke_catalyst_ is the coke (%wt.) loading in the used catalyst.

Coke yield on feed basis [coke_feed_ (%wt.)] was calculated based the following:

$${\mathrm{coke}}_{\mathrm{feed}}\left(\%wt\right)=(m_{\mathrm{coke}}\left(g\right)/mass\;of\;total\;feed\left(g\right))\times 100.$$ (2)

## Results and Discussion

### Catalyst Characterization

**FIGURE 1:**
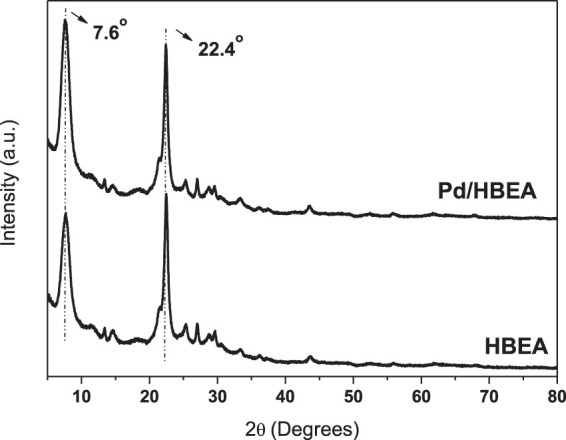
X-ray diffraction patterns obtained from HBEA and Pd/HBEA catalysts.

The elemental composition and textural properties of HBEA and Pd/HBEA catalysts are shown in Table 1. The introduction of metal slightly decreased the surface area, particularly the microporous area, compared to the external area. There was a slight decrease in porous volume after Pd introduction. These observations suggest that Pd is mainly located on the external surface of the BEA catalyst. X-ray diffraction patterns in Figure 1 could be considered very similar and suggest that impregnation of Pd does not affect the zeolite lattice. Moreover, no peak related to Pd was observed. DRX barely detects low concentrations and small metal particles.

**TABLE 1:** Elemental composition and textural properties of HBEA and Pd/HBEA catalysts.

Catalyst	Si/Al ratio	Pd (%w/w)	S_BET_ (m^2^. g^−1^)	S_Micro_ (m^2^. g^−1^)	S_ext_ (m^2^. g^−1^)	V_p_ (cm^3^. g^−1^)
HBEA	23	–	660	426	234	0.75
Pd/HBEA	23	0.85	582	372	210	0.71

**FIGURE 2:**
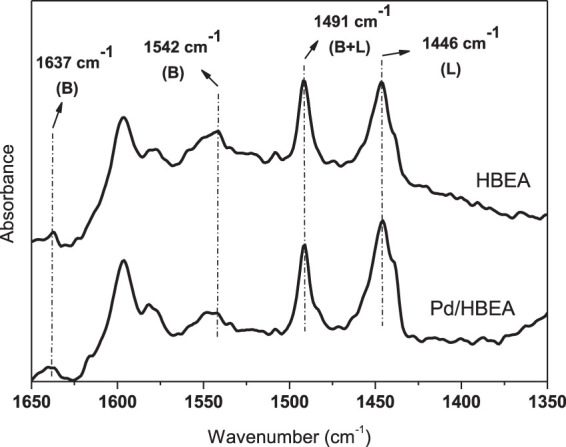
FTIR spectra obtained from adsorbed pyridine on HBEA and Pd/HBEA catalysts. Legend: B = Brønsted acid site; L = Lewis acid site; B + L = overlapping of peaks attributed to Lewis and Brønsted acid sites.

FTIR spectra from adsorbed pyridine were shown in Figure 2. Peaks at 1,637 cm^−1^ and 1,542 cm^−1^ are attributed to adsorbed protonated pyridine (pyridinium ion) on Brønsted acid sites. The peak at 1,491 cm^−1^ is attributed to the overlapping of peaks originated from pyridine interaction with Lewis and Brønsted acid sites. The peak at 1,446 cm^−1^ is attributed to the interaction of pyridine with the Lewis acid site (Anunziata et al., 2007; Zholobenko et al., 2020). In general, HBEA and Pd/HBEA showed similar acidic properties. However, after Pd loading, a slight decrease in the Brønsted acid site (1,542 cm^−1^) and a slight increase in the Lewis acid site (1,446 cm^−1^) were observed (peak deconvolution and peaks heights are described in detail in Supplementary Figure 2). Probably, during the Pd introduction, aluminum extra-framework species were formed and framework aluminum decreased. Furthermore, Pd may block some Brønsted sites of the catalyst.

### Selection of Initial Testing Conditions: Effect of Temperature and H_2_ Pressure on DX HDO

The hydrodeoxygenation (HDO) of DX results using three different experimental conditions are presented in Table 2.

**TABLE 2:** Product distribution obtained from DX HDO (10%DX = 10 g DX in 90 g n-hexane; 5 g catalyst; 40 bar of H_2_) carried out under different temperatures and reaction times.

Reaction time (h)	12		24		24	
Temperature (°C)	250		250		200	
	nA_6_	+ DX	nA_6_	+ DX	nA_6_	+ DX
DX conv. (%)	–	100	–	100	–	100
nA_6_ conv. (%)	13.1	0	35.7	0	5.0	0
**Yield (wt%)**						
Coke	<0.1	0.7	<0.1	0.6	<0.1	1.3
Gas	<0.1	0.5	0.4	0.4	<0.1	0.2
Organic liquid	94.7	91.6	94.3	90.0	94.4	91.2
Aqueous phase	–	3.8	–	3.8	–	3.8
Total	94.8	96.6	94.7	94.8	94.4	96.3
**Yield (wt%)**						
CO	–	0.1	–	0.1	–	<0.1
CO_2_	–	0.1	–	0.1	–	0.1
A_5-_	<0.1	2.0	1.4	2.2	0.3	0.7
Branched A_6_	13.0	0.3	34.3	0.3	0.7	0.3
CyAlk_6+_	<0.1	1.4	<0.1	1.2	<0.1	0.6
Alk_7+_	–	0.8	–	0.6	–	0.2
Oxygenates	–	0.1	–	0.1	–	0.7
Aromatics	–	0.1	–	0.1	–	0.1
Olefines	–	–	–	–	–	0.1
Non-identified	<0.1	0.6	<0.1	0.9	<0.1	1.5
CO_2_/CO	–	1	–	1	–	>1

A_5-_ = hydrocarbons with five or fewer carbons; branched A_6_ = branched hydrocarbons with six carbons; CyAlk_6+_ = cyclic hydrocarbons with six or more hydrocarbons; DX conv (%) = DX conversion (wt%); nA_6_ = n-hexane; nA_6_ conv. = nA_6_ conversion (wt%); Alk_7+_ = linear or branched hydrocarbons with seven or more hydrocarbons. For evaluation of n-hexane conversion, hydroconversion reactions were carried out using 90 g of pure n-hexane along with 5 g of catalyst.

In all experiments, the values of mass balance reached at least 94.8%. Hydrotreating n-hexane yielded a liquid phase where the major products were branched paraffins with six carbons such as 2,2-dimethyl-butane, 2-methyl-pentane, and 3-methyl-pentane. This was similarly observed for hydrotreating n-hexane in the presence of noble metal/HBEA in the literature (Lima et al., 2011; Saxena et al., 2013; Shimada et al., 2015).

No n-hexane conversion was observed when mixed with DX. In contrast, DX was fully converted in any reaction condition studied. The DX’s product distribution was profoundly affected by the reaction time and temperature. At 200°C, coke (and probable residues) and oxygenated compounds yields were the highest, whereas Alk_7+_ and CyAlk_6+_ yields were the lowest, compared to the other conditions. The reactions carried out at 250°C presented an increase in Alk_7+_ and CyAlk_6+_ yields and a decrease in coke yield; however, they resulted in a notable increase in A_5−_ yield. Most probably, the increase in reaction time from 12 to 24 h favored hydrogenolysis of Alk_7+_ and CyAlk_6+_ to lighter compounds such as A_5_. Thus, the condition of the reaction time of 12h and 250°C was chosen as a starting point for further H_2_ pressure investigation on DX HDO.

Hydrogen is the main and probably the most expensive feedstock used on hydrotreatment reactions. Besides, depending on reaction conditions, the excess of hydrogen could promote the transformation of heavier alkanes into lighter ones through hydrogenolysis. The effects of hydrogen pressures from 10 to 40 bar on the yield of different classes of compounds are shown in Table 3.

**TABLE 3:** Effect of hydrogen pressure on DX HDO of 10% (wt%) DX (10 g DX + 90 g n-hexane) and 5 g of Pd/HBEA for 12 h at 250°C.

Pressure (bar)	15		20		30		40	
	nA_6_	+ DX	nA_6_	+ DX	nA_6_	+ DX	nA_6_	+ DX
DX conv. (%)	–	100	–	100	–	100	–	100
nA_6_ conv. (%)	8.4	0	8.5	0	10.3	0	13.1	0
**Yield (wt%)**								
Coke	<0.1	1.3	<0.1	0.8	<0.1	0.8	<0.1	0.7
Gas	<0.1	0.4	<0.1	0.7	<0.1	0.6	<0.1	0.5
Organic liquid	95.5	91.2	97.0	90.9	97.2	89.3	94.7	91.6
Aqueous phase	–	3.8	–	3.8	–	3.8	–	3.8
Total	95.6	96.7	97.1	96.2	97.3	94.5	94.8	96.6
**Yield (wt%)**								
CO	–	0.1	–	0.2	–	0.1	–	0.1
CO_2_	–	0.1	–	0.2	–	0.2	–	0.1
A_5-_	<0.1	1.2	<0.1	1.9	<0.1	1.9	<0.1	2.0
Branched nA_6_	8.4	0.3	8.5	0.5	10.2	0.2	13.0	0.3
CyAlk_6+_	<0.1	0.7	<0.1	0.8	<0.1	1.2	<0.1	1.4
Alk_7+_	–	0.2	–	0.3	–	0.5	–	0.8
Oxygenates	–	0.5	–	0.1	–	0.2	–	0.1
Aromatics	–	0.1	–	0.3	–	0.3	–	0.1
Olefines	–	>0.1	–	>0.1	–	–	–	–
Not identified	<0.1	1.3	<0.1	0.8	<0.1	0.4	<0.1	0.6
CO_2_/CO	–	1	–	1	–	2	–	1

nA_6_ = n-hexane; DX conv (%) = DX conversion (wt%); nA_6_ conv. = nA_6_ conversion (wt%); A_5-_ = hydrocarbons with five or fewer carbons; branched A_6_ = branched hydrocarbons with six carbons; CyAlk_6+_ = cyclic hydrocarbons with six or more hydrocarbons; Alk_7+_ = linear or branched hydrocarbons with seven or more hydrocarbons. For evaluation of n-hexane conversion, hydroconversion reactions were carried out using 90 g of pure n-hexane along with 5 g of catalyst.

Conversion of pure n-hexane increased by increasing hydrogen pressure, and in all tests, n-hexane isomers were mainly produced. When the reactor was fed with a 10% DX/n-hexane solution, only DX was converted. These results suggest that DX hindered n-hexane conversion in all pressure ranges. Low coke yields were observed in all experiments containing a DX and n-hexane mixture, and as we expected, increasing hydrogen slightly decreases the coke yield. Lower amounts in CO and CO_2_ were observed. Furthermore, a low amount of oxygenates with the exception of oxygenates at 15 bar was observed.

DX produces a broad range of products, and only in the presence of DX, CyAlk_6+_ and Alk_7+_ were observed. More importantly, these products’ yields increased as hydrogen pressure increased. Oxygenates are presented in a lower amount and decreased by increasing pressure. However, there is also a simultaneous increase in A_5−_ yield with pressure. The other products, such as aromatics and olefins, were much smaller in yields and did not show significant changes with pressure. In general, at 40 bar, HDO affords slightly better products compared to 30 bar. Hence, we select an intermediary pressure (30 bar) for further studies.

### Catalyst/DX Ratio Effect on DX HDO

Data in Table 4 describe product distribution and yield obtained from reactions carried out at three different catalysts/DX ratios of 1/2, 1/1, and 2/1 (w/w).

**TABLE 4:** Effect of catalyst/DX ratio on DX HDO product distribution and yield (wt%). General reaction conditions: 250°C; 30 bar of H_2_; 12 h; 90 g n-hexane; catalyst/DX ratio (wt.): 1/2 = 5 g Cat/10 g DX; 1/1 = 10 g Cat/10 g DX; 2/1 = 10 g Cat/5 g DX.

Catalyst/DX ratio (wt/wt)	1/2	1/1	2/1	1/2	1/1	2/1
Feed	Mixtures	Mixtures	Mixtures	nA_6_	nA_6_	nA_6_
DX conv. (%)	100	100	100	–	–	–
nA_6_ conv. (%)	0	2.1	17.0	10.3	44.7	44.7
**Yield (wt%)**						
Coke	0.8	0.9	0.4	<0.1	<0.1	<0.1
Gas	0.6	0.9	0.4	<0.1	0.2	0.2
Organic liquid	89.3	89.3	92.1	97.2	96.3	96.3
Aqueous phase	3.8	3.8	2.0	–	–	–
Total	94.5	94.9	94.8	97.3	96.4	96.4
**Yield (wt%)**						
CO	0.1	0.2	0.0	–	–	–
CO_2_	0.2	0.2	0.1	–	–	–
A_5-_	1.9	1.8	1.4	<0.1	0.8	0.8
Branched A_6_	0.2	2.2	16.6	10.2	44.6	44.6
CyAlk_6+_	1.2	0.9	0.6	<0.1	<0.1	<0.1
Alk_7+_	0.5	0.5	0.4	–	–	–
Oxygenates	0.2	0.1	0.0	–	–	–
Aromatics	0.3	0.1	0.0	–	–	–
Olefines	–	<0.1	0.0	–	–	–
Non-identified	0.8	1.1	<0.1	<0.1	<0.1	<0.1
CO_2_/CO	2	1	>1	–	–	–

N-hexane conversion was extremely affected by changes in the catalyst mass. For example, a twofold increase in the catalyst mass increased fourfold the n-hexane conversion. As seen before, regardless of the catalyst/DX ratio applied, branched A_6_ compounds were still the major products of n-hexane conversion. Branched A_6_ yield and n-hexane conversion were quite similar. Therefore, since n-hexane is minorly converted into A_5−_ compounds and is majorly converted into its isomers, we can conclude that hydroisomerization is the main reaction taking place between n-hexane and Pd/HBEA catalyst.

Besides, when DX is used, A_5−_ and A_7+_ yields had a notable increase, suggesting that DX contributes to a range of compounds from A_5−_ to A_7+_. In particular, DX can yield n-hexane that could suffer hydroisomerization, yielding branched A_6_ compounds. However, an accurate contribution of DX and n-hexane to branched A6 compounds could not be determined. However, the branched A_6_ yields values that are close to the values of n-hexane conversion, suggesting that n-hexane is the main source of branched A_6_ compounds in experiments that DX/n-hexane mixtures were used.

The organic liquid was the major phase obtained from DX HDO, whatever the Cat/DX ratio applied. Coke yields were similar at the 1/2 ratio to those at the 1/1 Cat/DX ratio, whereas a significant decrease was observed at the 2/1 Cat/DX ratio. The gas-phase yield reached a peak at the 1/1 Cat/DX ratio.

**FIGURE 3:**
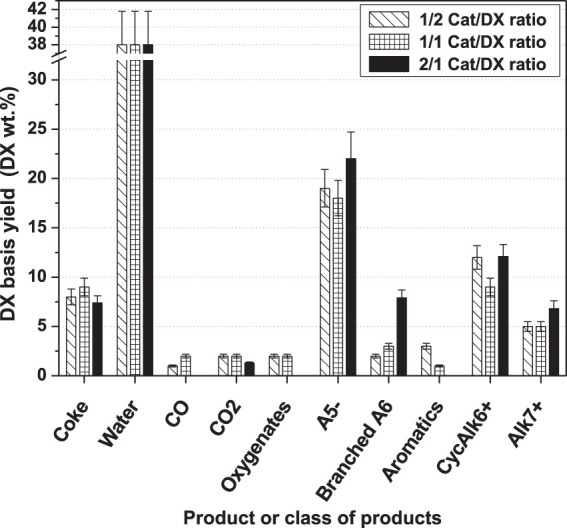
Effect of different Cat/DX ratios on products or a class of products yields calculated based on a DX basis (DX wt%).

The yields of all carbon-oxygenated compounds were negligible; therefore, we assume that water was the main product of DX hydrodeoxygenation. Since the amounts of DX were not the same in all cases, we recalculated product yields on a DX basis (wt%). Furthermore, we assumed no DX and n-hexane interaction. With this assumption, we discounted from the DX products the n-hexane contribution, i.e., branched A_6_ and A_5−_ (the only class of compounds produced by n-hexane) based on its conversion as presented in Figure 3.

Data illustrated in Figure 3 show two important features of the 2/1 Cat/DX ratio. Firstly, it enhances the hydrodeoxygenation of DX as oxygenates were not observed. Here, one could also emphasize a further advantage of the 2/1 Cat/DX ratio to improve DX deoxygenation mainly into the water, as CO was not observed and CO_2_ yield diminished. This endorses the improvement in transferring carbon atoms from DX to hydrocarbons. Secondly, it increases the yield of some hydrocarbon classes such as A_5−_ (slightly) branched A_6_, and Alk_7+_. However, the CycAlk_6+_ yield is similar to that of the other Cat/DX ratios.

However, the large amount of A_5−_ compounds may indicate inappropriate reaction time, leading to excessive parallel reaction, like sugar intermediate or hydrogenolysis of the hydrocarbon products. Hence, we investigated the effect of reaction time using the 2/1 Cat/DX ratio next.

### Effect of Time on DX Hydroconversion

**FIGURE 4:**
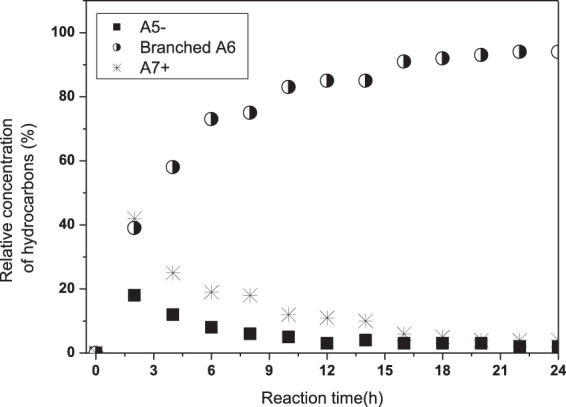
Relative concentration of some products, in the liquid phase, obtained from 5 g DX/90 g n-hexane using 10 g Pd/HBEA at 250°C under 30 bar pf H_2_ for 24 h. A5− = alkanes with five or fewer carbons. Branched A6 = branched alkanes with six carbons; A7+ = alkanes with seven or more carbons.

**FIGURE 5:**
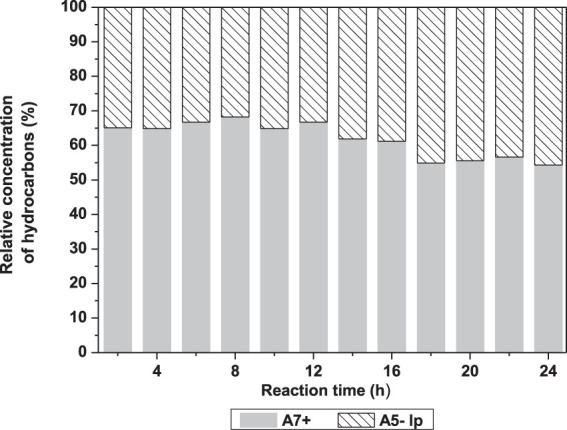
Composition of alkanes with five or fewer carbons (A_5-_ lp, **Panel A**) and alkanes with seven or more carbons (A_7+_, **Panel B**), including methyl cyclopentane and cyclohexane, over reaction time in the liquid phase.

The effect of the reaction time was first explored by withdrawing aliquots of liquid products at time intervals of 2 h and up to 24 h. Figure 4 illustrates the relative yields obtained from three classes of hydrocarbon products as oxygenated compounds were fully converted after 4 h. Further, in order to avoid interference of the high concentration of A_6_ isomers, almost exclusively from n-hexane, we presented in Figure 5 the relative composition of alkanes with five or fewer carbons (A_5−_) and alkanes with seven or more carbons (A_7+_), including methyl cyclopentane and cyclohexane, in a mixture where A_6_ was not included. Figure 5 shows the increase in A_5−_ and decrease in A_7+_ hydrocarbons with increasing the reaction time. Considering no oxygenates were observed after 4 h of reaction, light hydrocarbons, A_5−_, have an important contribution from hydrogenolysis of A_7+_ products.

Further, to conduct a complete analysis of all products for the transformation in batch mode, we carried out the reaction at selected reaction times. As shown in Table 5, the material balance achieved values above 92% (wt%). The liquid phase was reconfirmed as the main product, and DX was fully converted in all experiments. N-hexane conversion pure and its mixture with DX are presented in Supplementary Figure 3. In the presence of DX, n-hexane conversion was very small up to 4 h and then has a step jump to a significantly higher value, but still less than the conversion with pure feed at the corresponding time. These results are probably related to n-hexane being less competitive to acid and metallic sites, not only with DX but also among other intermediate products. Pure n-hexane produced mainly branched A_6_. Increasing reaction time increased both n-hexane conversion and branched A_6_, and only a slight amount of A_5−_ was observed. Oxygenate compounds were negligible at 2 h and were almost completely converted into hydrocarbons at 4 h, as already observed in the experiments in Figure 3. Hence, DX and oxygenates are converted more readily before n-hexane. Further, larger hydrocarbon produced from DX (CyAlk_6+_ and Alk_7+_) may compete with n-hexane to active sites. Thus, the profile of Supplementary Figure 3 should be rationalized by a competition to acid sites, suggesting a reactivity of the following order: DX > oxygenates > CyAlk_6+_ and Alk_7+_ > n-hexane.

**TABLE 5:** Effect of reaction time on DX HDO product distribution and yield (wt%). Conditions: 250°C; 30 bar of H_2_; 90 g n-hexane; 5 g DX; 10 g Cat; catalyst/DX = 2/1.

Reaction time (h)	2	4	8	12	2	4	8	12
	nA_6_	nA_6_	nA_6_	nA_6_	+ DX	+ DX	+ DX	+ DX
DX conv. (%)	–	–	–	–	100	100	100	100
nA_6_ conv. (%)	3.1	15.6	34.2	44.7	2.0	2.1	19.8	17.0
**Yield (wt%)**								
Coke	<0.1	<0.1	<0.1	<0.1	0.5	0.4	0.3	0.3
Gas	<0.1	<0.1	0.2	0.2	0.3	0.4	0.5	0.4
Organic liquid	93.3	94.1	94.4	96.3	92.4	89.7	89.5	92.1
Aqueous phase	–	–	–	–	2.0	2.0	2.0	2.0
Total	93.4	94.2	94.5	96.4	95.2	92.5	92.3	94.8
**Yield (wt%)**								
CO	–	–	–	–	<0.1	<0.1	0.0	0.0
CO_2_	–	–	–	–	<0.1	0.1	0.1	0.1
A_5-_	<0.1	<0.1	0.8	0.8	1.1	1.0	1.4	1.4
Branched A_6_	3.1	15.6	33.3	44.6	2.4	2.6	18.5	16.6
CyAlk_6+_	<0.1	<0.1	<0.1	<0.1	0.6	0.6	0.8	0.6
Alk_7+_	–	–	–	–	0.3	0.3	0.3	0.4
Oxygenates	–	–	–	–	<0.1	<0.1	0.0	0.0
Aromatics	–	–	–	–	<0.1	<0.1	<0.1	0.0
Olefines	–	–	–	–	–	–	0.0	0.0
Non-identified	<0.1	<0.1	<0.1	<0.1	0.7	0.7	<0.1	<0.1
A_5-_/A_7+_	–	–	–	–	1.2	1.1	1.3	1.4

A_5-_/A_7+_ = quotient between A_5-_ and A_7+_ yields; A_7+_ yield = summing up of CyAlk_6+_ and Alk_7+_ yields.

The results showed that A_5−_ yield increased with an increase in reaction time. The A_5−_/A_7+_ ratio indicated this trend even more clearly. In this context, it worth noting the advantage achieved by decreasing reaction time to increase yield in CyAlk_6+_ and Alk_7+_ products. This will be further discussed in the next section. Coke yield showed a slight decrease at a longer reaction time. In this work, residues in the catalyst from DX are generally related to coke, and they also include condensed products, which may sequentially react and be removed with time. In this context, it should be pointed out that even at the lowest hydrogen pressure applied (15 bar) as presented in Table 3, coke yield was low and that catalyst was still capable of yielding CyAlk_6+_ and Alk_7+_. Most probably, the lower hydrogen available (at a low pressure) is mainly responsible for oxygenates and lower CyAlk_6+_ and Alk_7+_ yields.

### Considerations and Insights Into Reactions Pathways

**FIGURE 6:**
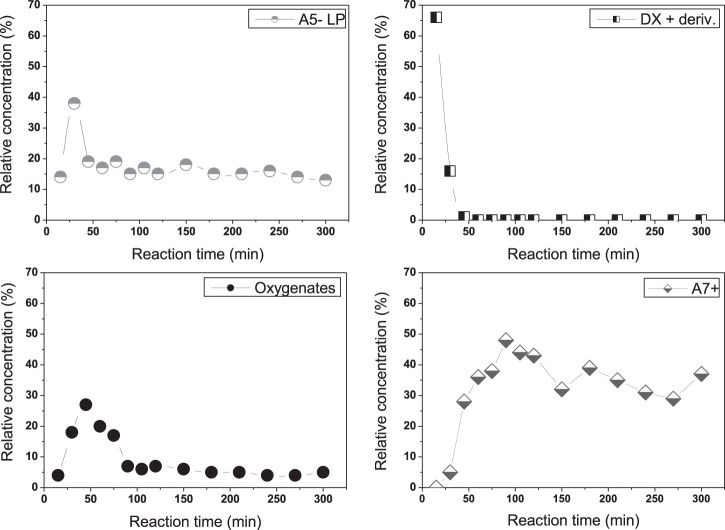
Relative concentrations of different classes of products (% of total carbon of the liquid) obtained from the hydroconversion of 5 g of DX in 90 g of n-hexane using 10 g of Pd/HBEA at 250°C under 30 bar of H_2_. Aliquots were withdrawn from the liquid phase at time intervals of 15 min. A5− LP = alkane with five or fewer carbons in liquid phase; A7+ = alkane with seven or more carbons; DX + deriv. = DX derivatives (ketal carbohydrates containing four and five oxygens); oxygenates = oxygenated products (mainly mono-oxygenated compounds like ketones, furfurals, and alcohols).

In order to unveil the compounds involved in DX transformation into alkanes, the time of withdrawal of aliquotes from the liquid phase was further refined to every 15 min for the first 5 h. The data are displayed in Figure 6 and the identification of main compounds and their relative concentrations is presented in Supplementary Table 1.

In the first 50 min, DX and some carbohydrates derived from it (containing in their structures different functional groups) could be detected, although their relative concentrations fell from 55 to 0%. In this period, the relative concentrations of A_5−_ and oxygenates reached their peaks at 25 and 50 min, respectively. Some works (Deneyer et al., 2018; Jin et al., 2019) have shown hydrodeoxygenation of pentose and hexoses to butanes and pentanes and less carbon-chain hydrocarbons. Thus, this strongly indicates that xylose and acetone, yielded by a reverse DX reaction, probably contributed to the total A_5−_ compounds. After 100 min, A_5−_ and oxygenates decreased and remained constant until the end of the period. A_7+_ selectivity presented a continuous increase from 15 to 100 min and then a low decrease. Thus, an additional reaction pathway contributes to A_5−_ production, the most probable hydrogenolysis of larger hydrocarbons.

Further information was also obtained from our previous work (Batalha et al., 2014). It was demonstrated that DX (labeled as ^13^C in xylofuranose) produces a broad range of ^13^C in a hydrocarbon containing seven or more carbons. Thus, both xylofuranose and isopropylidene contribute to the oxygenates pool.

**FIGURE 7:**
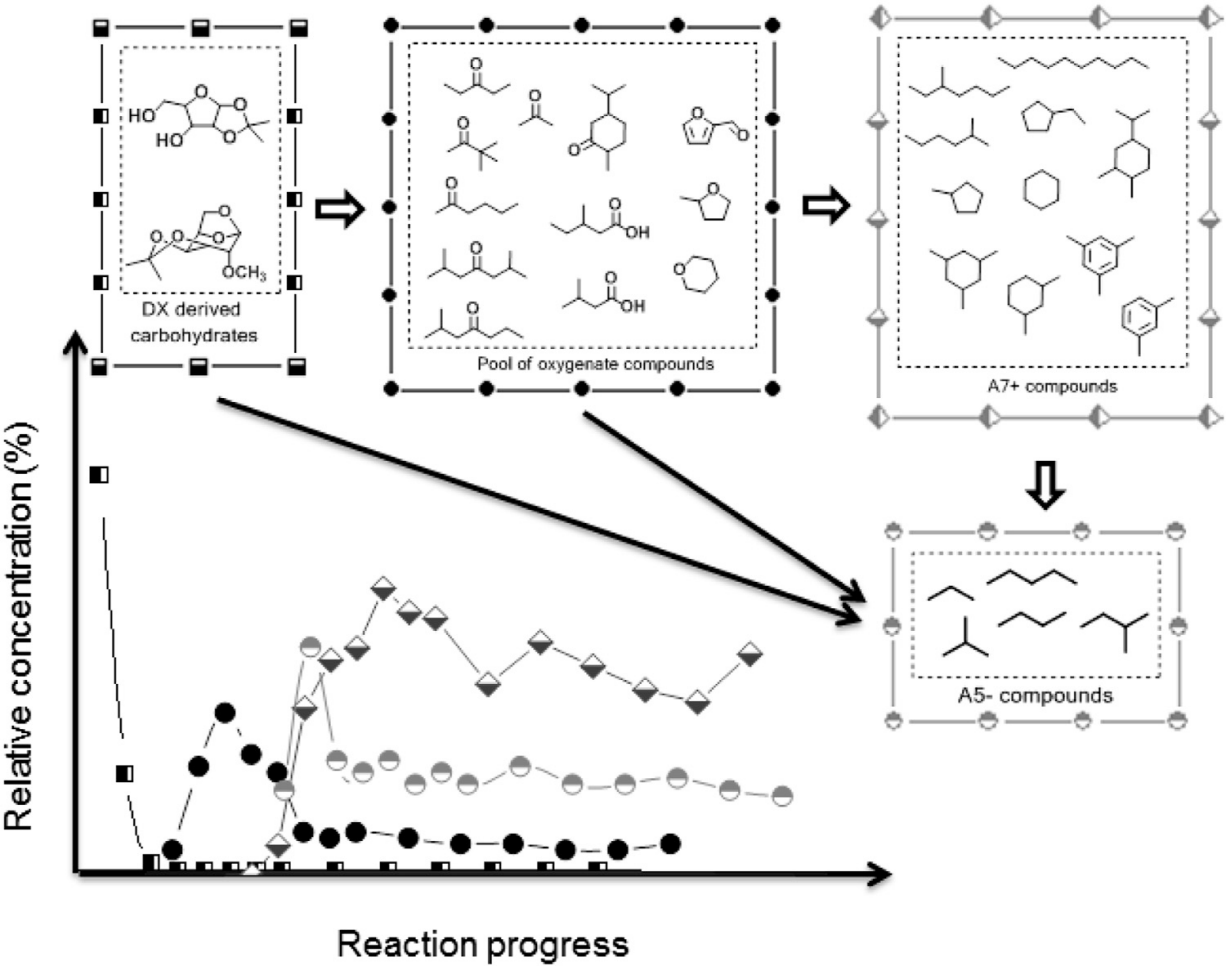
Insight into DX HDO pathways and main compounds involved in these reactions.

Considering those previous results, we proposed a scheme illustrated in Figure 7, containing the major compounds produced through DX HDO and reaction pathways undergone by these compounds. While a detailed mechanistic insight of the DX reactions into the observed hydrocarbons is not possible at the moment, the results presented herein allow us to establish a sequence of events with detected intermediates at different reaction times, as illustrated in Figure 7. This figure contains the major compounds produced through DX HDO.

As shown from the analysis of the liquid products after the first period of time on stream, the DX transformation begins through a partial deketalization and dehydration, yielding xylose monoketal and anhydro-carbohydrate ketal, respectively. Xylose could not be detected due to low solubility in the organic phase. Then, these compounds can yield A_5−_ compounds along with a pool of oxygenate compounds (a wide variety of mono-oxygen compounds, which are majorly ketones, furfurals, and alcohols; see Supplementary Table 1). Due to the well-known textbook reactivity of these oxygenated functions, this pool of oxygenates can undergo condensation, hydrodeoxygenation, cyclization, and aromatization reactions yielding A_7+_ compounds, including naphthenes. Finally, heavy hydrocarbons can still be cracked by acid sites or undergo hydrogenolysis by metallic sites yielding A_5−_ compounds.

### Comparison of Different Pd-Zeolite Catalysts on HDO DX

In our previous work (Pereira et al., 2021), a pristine HZSM-5 was impregnated with Pd and its performance on DX HDO reactions in a batch reactor was investigated. This section evaluates an HZSM-5-type catalyst (Pd/HZSM-5) performance in an optimized DX HDO reaction condition used by Pd/HBEA. The results are presented in Table 6.

**TABLE 6:** Comparison between Pd/HZSM-5 and Pd/HBEA catalysts on DX HDO product distribution and yield (wt%). Conditions: 250°C; 30 bar of H_2_; 90 g n-hexane; 5 g DX; 10 g Cat; catalyst/DX = 2/1; reaction time = 4 h.

Catalyst	Pd/HZSM-5	Pd/HBEA	Pd/HZSM-5	Pd/HBEA
Si/Al ratio	23	23	23	23
SBET (m^2^/g)	394	645	394	645
Pd loading (%)	0.52	0.85	0.52	0.85
**Feed**	**Mixtures**	**Mixtures**	**nA6**	**nA_6_**
DX conv. (%)	100	100	–	–
nA6 conv. (%)	<1.0	2.1	30.7	15.6
**Yield (wt%)**				
Coke	0.6	0.4	<0.1	<0.1
Gas	0.6	0.4	0.3	<0.1
Organic liquid	93.4	89.7	94.2	94.1
Aq. Phase	2.0	2.0	–	–
Total	96.6	92.5	94.5	94.2
**Yield (wt%)**				
CO	0.0	<0.1	–	–
CO_2_	0.1	0.1	–	–
A5-	1.1	1.0	0.8	<0.1
Branched A6	0.5	2.6	27.8	15.6
CyAlk6+	0.3	0.6	<0.1	<0.1
Alk7+	0.1	0.3	<0.1	–
Oxygenates	0.2	<0.1	–	–
Aromatics	<0.1	<0.1	–	–
Olefines	–	–	–	–
Non-identified	0.1	<0.1	<0.1	<0.1
CO_2_/CO	>0.1	>0.1		

*Further details about Pd/HZSM-5 catalyst and its characterization data can be found elsewhere (Pereira et al., 2021).

When pristine n-hexane was used, the liquid fraction was the major product and the coke yield was negligible. However, more gas products were obtained when the reaction was carried out using Pd/HZSM-5 catalyst. These observations were consistent with the cracking properties of ZSM-5 zeolite vs. beta zeolite.

When the reaction was carried out using DX/n-hexane mixture, material balance attained values above 92%, and organic liquid fractions remained the major component. Pd/HZSM-5 zeolites yielded more coke and gas than Pd/HBEA; moreover, Pd/HZSM-5 catalysts yielded more oxygenated compounds. Unlike Pd/HBEA catalysts, Pd/HZSM-5 catalysts could not effectively convert n-hexane when mixed with DX and afford higher oxygenates yield. The Pd/HBEA catalyst provided a more than twofold increase in CyAlk_6+_ and Alk_7+_ yields compared to the Pd/HZSM-5 catalyst. Both catalysts yielded almost the same A_5−_ quantity.

Branched A_6_ compounds yield was more accentuated when Pd/HBEA was used. This could be interpreted as the product of n-hexane caused by higher accessibility. Experiments carried out using only pristine n-hexane as feed presented liquid fraction as the main product with branched A_6_ compounds as the principal component.

Thus, the results demonstrated that the Pd/HBEA catalyst was capable of enhancing DX conversion into hydrocarbons by, first, improving DX deoxygenating and, second, yielding more A_7+_ (CyAlk_6+_ and Alk_7+_) compounds compared with the Pd/HZSM-5 catalyst. These results may be first rationalized based on larger pore size and higher external area of Pd/HBEA. The latter should favor primary DX products. Then, the larger pore size may also facilitate reactions such as condensation, which favors the formation of A_7+_ compounds compared to the Pd/HZSM-5 catalyst.

## Conclusion

This work demonstrates the production of high values of green alkanes using a model compound, DX. Pd/HBEA showed good potential to hydrodeoxygenation of DX using a batch reactor. First, we demonstrated the importance of some selection key reaction parameters: high catalyst/DX ratio and hydrogen partial pressure favored deoxygenation and decreased coke yield. However, long reaction times favor sequential reactions of heavier alkanes (containing seven or more carbons = A_7+_), giving lighter alkanes (containing five or fewer carbons = A_5−_). Using optimized reaction parameters (10 g Pd/HBEA mixed with 5 g DX/90 g n-hexane for 4 h under 30 bar of hydrogen at 250°C), we achieved full DX deoxygenation, low coke yield of 6%*p*/*p* of DX, and an A_5−_/A_7+_ ratio around 1. Moreover, Pd/HBEA showed higher deoxygenation, higher selectivity to target produces, and less coke yield than Pd/ZSM-5.

Our results demonstrated a competition for active sites in the following order: DX > oxygenates > larger hydrocarbons > n-hexane. For instance, in a low catalyst/DX ratio of 1/2, no n-hexane conversion was observed, while DX was fully converted. In general, n-hexane conversion was much lower in the presence of DX compared to pure n-hexane. Further, from the profile of products as a function of the reaction time, the disappearance of oxygenates and A_7+_ formation, for example, these compounds are considered intermediate between DX and n-hexane in the competition for active sites.

Through reaction monitoring, the formation of a pool of oxygenated compounds such as furans, ketones, carboxylic acids, and lighter and heavier alkanes was observed. Based on the literature, insights into hydrocarbons production could be proposed. Preferred products such as cycle-alkane and paraffin containing seven or more carbons (A_7+_) are produced from mainly secondary oxygenates intermediates (undergoing condensation, cyclization, and hydrogenation reactions). Light hydrocarbons (A_5−_) should be produced from hydrogenolysis of A_7+_ hydrocarbon and direct hydrogenation of decomposition product of DX.

## Data Availability

The original contributions presented in the study are included in the article/supplementary material; further inquiries can be directed to the corresponding author.
