# Supplementary material for: Hydrodeoxygenation of Xylose Isopropylidene Ketal Over Pd/HBEA Catalyst for the Production of Green Fuels
Source: Front Chem. 2021 Aug 20;9:729787. doi: 10.3389/fchem.2021.729787 (PMC8417725; doi:10.3389/fchem.2021.729787)
Supplement: Supplementary file 1 [file DataSheet1.PDF]

Supplementary material

Hydrodeoxygenation of xylose isopropylidene ketal over Pd/HBEA catalyst for production of green fuels

Souza, M.O.[1]; Pereira, S.F.C.[2], Lam, Y.L.[1], Soter, L.M.M.[1]; Pereira, M.M.[1*,2]

[1] Instituto de Química, Universidade Federal do Rio de Janeiro, Rio de Janeiro, RJ, Brazil
[2] Instituto de Química, Universidade do Estado do Rio de Janeiro, Rio de Janeiro, RJ, Brazil

EXPERIMENTAL SECTION

Qualitative and quantitative analyses of liquid products

Some liquid samples produced from DX HDO reactions could contain up to 130 different compounds. Considering this, 9 standard compounds purchased (heptane, benzene, toluene, o-xylene, 1,3,5-trimethyl benzene, cyclohexane, methyl cyclohexane, 1,2-dimethyl cyclohexane and 3-pentanone) were selected to assure an accurate identification and quantitation analyses. Moreover, these compounds were assigned to different classes (paraffins, naphtenes, aromatics and oxygenated organic compounds) in order to facilitated data treatment and understanding of work, as well. In this sense, standard compounds' retention times along with compound identification provided by NIST library mass spectra database were used to perform the qualitative analysis. Quantitative analysis was performed through external standard calibration using standard compound response factors obtained from flame ionization detector. For example, concentration of toluene was calculated from toluene area and toluene calibration curve. P-xylene or m-xylene concentrations were calculated through o-xylene's curve whereas concentration of cycloalkanes with 8 or more carbons were calculated through 1,2-dimethy-cyclohexane's curve. Paraffins were quantitated through heptane's curve. Oxygenated compounds on liquid fraction were quantitated through calibration curve produced by 3-pentanone as standard compound . N-hexadecane was used as internal standard. Standard compounds calibration curve was obtained through dissolution of standard compounds in n-hexane on concentrations varying from 0,1mg/mL to 1,4mg/mL concentration of n-hexadecane was fixed on 0.7mg/mL. Sample preparation consisted of dissolution of 20µL of internal standard solution (50mg/mL of n-hexadecane/n-hexane) into 1000µL of liquid product fraction. All the standard compounds were purchased from Sigma-Aldrich.

For determination of n-hexane conversion, calibration curve was obtained through dissolution of n-hexane (standard compound) and n-hexadecane (internal standard) into acetone. Concentration of n-hexadecane was equal to 0.7mg/mL and concentration of n-hexane varies from 0.4mg/mL to 1.2mg/mL. For sample preparation, 100µL of n-hexadecane along with 100µL of liquid product fraction were dissolved into 900 µL of acetone.

Below are described some equations adopted for data treatment.

Compound group relative concentration (R_{conc} (%)) was calculated through Equation S1:

Equation S1 $$R_{conc}(\%) = (\sum A_{products\ group} / \sum A_{total\ products}) \times 100$$

Where,

$\sum A_{products\ group}$ = summing up of absolute areas of products related to a group

$\sum A_{total\ products}$ = summing up of absolute areas of all products

Mass of product ($m_{product}$) in Iiquid fraction was calculated through Equation S2.

Product distribuition ($\%_{product}$) in liquid products was calculated through Equation S3. Equation S4 describes product yield in liquid product fraction.

Equation S2 $$m_{product}\ i\ (g) = C_{product}\ i \times \text{mass of liquid product fraction}$$

Equation S3 $$\%_{product}\ i\ (\%) = (m_{product}\ i / \sum m_{products}) \times 100$$

Where,

Equation S4 $$Y_{product}\ i\ (\text{wt. }\%) = (m_{product}\ i / \text{mass of total feed}) \times 100$$

$C_{product}\ i$ = concentration of product i in liquid product fraction

$\sum m_{products}$ = summing of mass products in liquid product fraction

Quantitative and qualitative analyses of gaseous products

As described on manuscript, the quantitative analysis of gaseous products used standard compound molar response factors (MRF) and their respective retention times along with brine-shift mass. A scheme of this apparatus is illustrated on Figure S1.

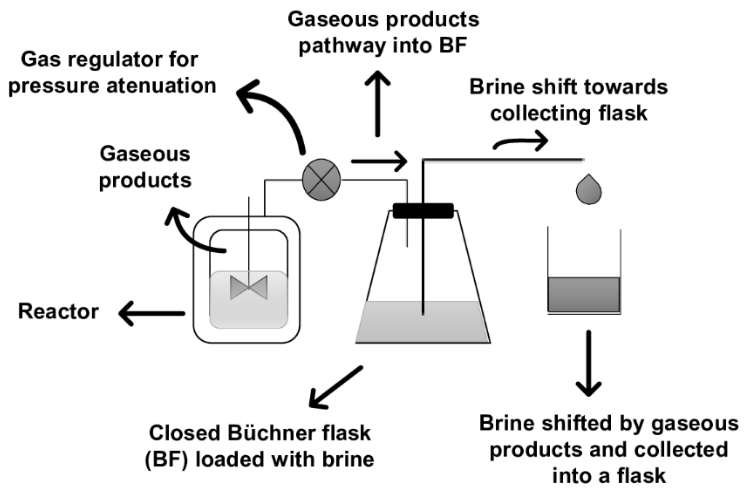

Supplementary Figure 1 | Brine-shift apparatus used in quantitative analysis of gaseous products of DX HDO reactions

It was assumed that gas inside Erlenmeyer was in ideal conditions. Retention times along with molar response factors (MRF) of standard compounds were used to identify and quantitate, respectively, different compounds on gaseous products. Gaseous product masses were calculated using the equations S5, S6 and S7.

Gaseous product moles were calculated following Equation 5.

Where,

n_{total} = total of gaseous product moles collected into BF

Equation S5 n_{total} (mol) = [p x (m_{brine} / d_{brine})] / (R x T)

m_{brine} = brine mass shifted by gaseous products
d_{brine} = brine density
R = 0.082 L x atm x K^{-1} x mol^{-1}
T = 298 K
p = 1 atm

Molar fraction of gaseous product i on gas collected into BF (% $n_{product}$ i) were calculated from Equation 6.

Equation S6 % $n_{product}$ i = Area $_{gas\ i}$ / MRF gas i

Where,

Area $_{gas}$ i = Area of gas i

MRF $_{gas}$ i = Molar response factor of gas i

Finally, mass of gas i was obtained from Equation S7.

Equation S7 $$m_{gas}\ i\ (g) = \%\ n_{product}\ i \times n_{total} \times MW_{gas}\ i$$

Where,

MW_{gas} i = molecular weight of gas i

RESULTS AND DISCUSSION: ADDITIONAL DATA

Compounds produced through DX HDO

In Supplementary Table 1 are described the most commonly compounds yielded from HDO DX reactions.

Supplementary Table 1 | Most commonly compounds and their relative concentrations (% of total carbon of the liquid products) obtained from the hydroconversion of 5 g of DX in 90 g of n-hexane using 10 g of Pd/HBEA at 250°C under 30bar of H_2. Results obtained from aliquots withdrew from liquid phase at 30, 45 and 60 min. A5- = Alkane with 5 or less carbons; A7+ = Alkane with 7 or more carbons; DX D. = DX-derivatives (ketal carbohydrates derived from DX); Oxyg. = oxygenate products; B. A6 = Branched alkanes with 6 carbons.

		Reaction time			
	Compound	30 min	45 min	60 min	
	Propane	0.8	1.1	1.3	
	Isobutane	2.4	5.4	6.5	
A5-	Butane	0.2	0.5	0.7	R
	2-Methyl-Butane	32.1	9.5	6.4	E
	Pentane	0.9	2.1	2.3	L
	2,2-Dimethyl-Butane	0.0	0.1	0.2	A
B. A6	2-Methyl-Pentane	1.4	4.8	7.1	T
	3-Methyl-Pentane	20.9	18.9	18.9	I
	Acetone	0.2	0.1	0.0	V
	3-Pentanone	0.7	1.3	0.4	E
	2,3 Dimethyl butanone	0.1	0.0	0.0	
	Methyl Isobutyl Ketone	7.4	12.0	8.9	
	Cyclopentanone	0.5	0.0	0.0	C
	2-Hexanone	0.2	0.2	0.2	O
	4-Methyl-3-Penten-2-one,	0.3	0.0	0.0	N
	4-Hydroxy-4-Methyl-2-Pentanone	0.9	0.0	0.0	C
	Butyrolactone	0.3	0.0	0.0	E
	3-Heptanone	0.2	0.6	0.0	N
	3-Heptanone	0.2	0.0	0.0	T
	4-Octanone	0.1	0.1	0.4	R
	2-Octanone	0.1	0.0	0.1	A
Oxyg.	4,6-Dimethyl-2-Heptanone	0.2	2.1	2.2	T
	4,6-Dimethyl-2-Heptanone	0.0	0.7	0.9	I

	2-(1-Methylpropyl)-Cyclopentanone	0.2	0.4	0.0	O
	1-Cyclopentyl-2-Propanone	0.1	0.0	0.0	N
	2-Methyl-2-Nonen-4-one	0.2	0.0	0.2	
	5-Decanone	0.2	0.0	0.3	(%)
	2-Dodecanone	0.2	0.0	0.0	
	3-Methyl-Butanoic acid	0.0	0.5	0.5	
	3-Methyl-Pentanoic acid	0.0	1.2	0.0	
	Furfural	0.6	0.0	0.0	
	2-Methyl- Tetrahydrofuran	0.0	0.0	0.0	
	Dihydro-2H-Pyran-3(4H)-one	0.1	0.0	0.0	
	1-Heptanol	0.1	0.0	0.0	
	Tetrahydropyran	0.3	0.6	0.0	
	Dihydro-2-Methyl-3(2H)-Furanone	0.0	0.0	0.0	
Dx D.	1,2:3,5-bis-*O*-(1-methylethylidene)-α-D-Xylofuranose	13.9	0.6	0.2	
	1,6-Anhydro-3,4-*O*-isopropylidene-2-*O*-methyl-β-D-galactopyranose	0.5	0.0	0.0	R
	1,2-*O*-isopropylidene-α-D-xylofuranose	0.1	0.0	0.0	E
	2,4-Dimethyl-Pentane	0.5	1.0	1.4	L
	3,4-Dimethyl-Pentane	0.0	0.1	0.3	A
	2-Methyl Hexane	0.0	0.9	0.3	T
	3-Methyl-Hexane	0.0	0.0	0.9	I
	2,3-Dimethyl-Pentane	0.0	0.6	0.7	V
	1,1-Dimethyl-Cyclopentane	0.1	0.6	0.1	E
	3-Methyl-Hexane	0.0	0.0	0.5	
	1,3-Dimethyl-, cis-Cyclopentane	0.0	0.0	0.3	
	1,3-Dimethyl-Cyclopentane	0.0	0.0	0.2	C
	Isopropylcyclobutane	0.0	0.0	0.2	O
	Heptane	0.0	0.7	0.8	N
	Ethyl-Cyclopentane	0.0	0.3	0.4	C
	2,4-Dimethyl-Hexane	0.0	0.2	0.2	E
	1,2,4-trimethyl-Cyclopentane and their isomers	0.0	0.2	0.3	N
	2-Methyl-Heptane and their isomers	0.1	1.8	2.0	T
	1,3-Dimethyl-Cyclohexane and their isomers	0.8	1.4	1.4	R
	1-Ethyl-3-Methyl-Cyclopentane and their isomers	1.0	0.5	0.5	A
A7+	Octane	0.0	0.6	0.5	T
	1,3-Dimethyl-, trans-Cyclohexane	0.0	0.5	0.5	I
	(1-Methylethyl)-Cyclopentane	0.0	0.3	0.3	O
	1,1,3-Trimethyl-Cyclohexane and their isomers	0.10	8.40	11.70	N
	1,3-Dimethyl-Benzene	0.0	0.1	0.1	
	Nonane	0.0	0.3	0.4	(%)
	1-Ethyl-4-Methyl-trans-Cyclohexane,	0.0	0.0	0.1	

	1-Methyl-2-Propyl-Cyclopentane	0.0	0.0	0.1	R
	Octahydro-2-Methyl-Pentalene	0.0	0.0	0.2	
	(1-methylethyl)-Cyclohexane	0.0	0.0	0.2	
	Propyl-Cyclohexane	0.0	0.7	0.7	
	Butyl-Cyclopentane	0.2	0.2	0.2	
	3,4-Dimethyl-Heptane	0.0	0.0	0.2	
	4-Methyl-Nonane	0.0	0.3	0.3	
	2-Methyl-Nonane	0.0	0.2	0.2	
	Mesitylene	0.0	0.0	0.3	
	1-Methyl-4-(1-Methylethyl)-, cis-Cyclohexane	0.0	0.5	0.6	
	1-Methyl-3-(2-Methylpropyl)-Cyclopentane	0.0	0.8	0.8	R
	1,2-Diethyl-cis-Cyclohexane	0.0	0.1	0.1	E
	1-Methyl-2-propyl-Cyclohexane	0.3	0.2	0.2	L
	1-Methyl-3-propyl- Cyclohexane	0.0	0.4	0.3	A
	Decane	0.2	0.4	0.4	T
	1-Methyl-4-(1-methylethyl)-Cyclohexane	0.0	0.0	0.1	I
	1,2-Dimethyl-3-(1-Methylethyl)-Cyclopentane	0.0	0.1	0.1	V
	1,1'-(1,2-Dimethyl-1,2-Ethanediyl)bis-, (R*,R*)-(.+/-.)-Cyclohexane,	0.0	0.2	0.3	E
	3,5-Dimethyl-Cyclohexanol	0.0	0.0	0.2	
	Butyl-Cyclohexane	0.2	0.4	0.5	
	1-Methyl-4-(2-Hydroxyethyl)-Cyclohexane	0.0	0.1	0.1	C
	1-Methyl-2-Methylene cyclohexane	0.0	0.2	0.1	O
	Decahydro-trans-Naphthalene	0.0	0.4	0.2	N
	(2-Methylpropyl)-Cyclohexane	0.0	0.0	0.3	C
	1-Ethyl-4-Methylcyclohexane	0.0	0.0	0.2	E
	1,4-Dimethyl-2-Octadecyl-Cyclohexane	0.0	0.0	0.3	N
	1-Ethyl-2-Propyl-Cyclohexane	0.0	0.2	0.1	T
	2-Methyl-Trans-Decalin	0.0	0.3	0.3	R
	Decahydro-2-Methyl-Naphthalene	0.0	0.0	0.4	A
	1-Methyldecahydronaphthalene	0.0	0.0	0.2	T
	Pentyl-Cyclohexane	0.0	0.0	0.3	I
	Decahydro-2,3-Dimethyl-Naphthalene	0.0	0.0	0.1	O
	1,4-Dimethyl-2-(2-Methylpropyl)-Benzene	0.0	0.3	0.3	N
	1-Methyl-2-Pentyl-Cyclohexane	0.0	0.0	0.4	
	1-Methyl-4-(1-methylbutyl)-Cyclohexane	0.0	0.0	0.1	(%)
	2-Ethyldecahydro-Naphthalene	0.0	0.0	0.2	
	2,3-Dihydro-4,7-Dimethyl-1H-Indene	0.0	0.0	0.2	
	(3-Methyl-2-Butenyl)-Benzene	0.0	0.0	0.2	

	1-Methyl-4-(1-methylbutyl)-Cyclohexane	0.0	0.0	0.2	

Since DX HDO reactions could yield a wide range of compounds (more than 150 in some experiments), we chose to arrange them in class of compounds in order to ease the results discussion in manuscript. However, we present identification of the major and most important molecules attributed to each class of compounds along with their relative concentration (%) in Supplementary Table 1. These results are part of the total molecules used to discuss some the section about insights of reaction pathways described in manuscript (Figures 6 and 7).

Evaluation of change on catalytic acid sites through FTIR spectra from adsorbed pyridine

On Figure S2 are depicted two FTIR spectra obtained after pyridine adsorption in HBEA and Pd/HBEA

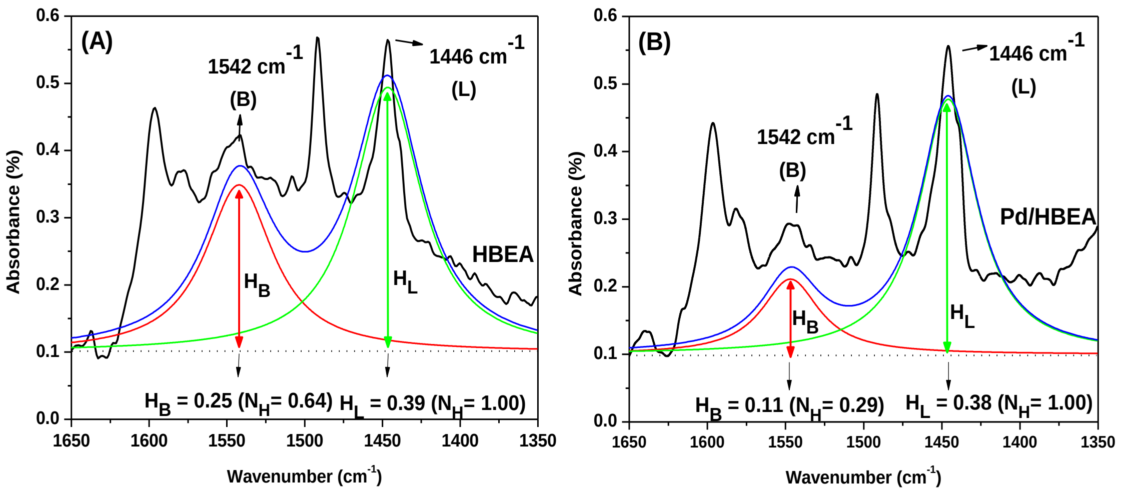

Supplementary Figure 2 - FTIR spectra obtained after pyridine adsorption in HBEA (A) and Pd/HBEA (B); N_H = normalized height. B = Brønsted acid site; L = Lewis acid site.

Deconvolution and integration of FTIR peaks related to specific Brønsted and Lewis acid sites (1542 and 1446 cm^{-1}, respectively) demonstrated the changing on Bronsted/Lewis acid sites balance on catalyst after Pd loading. After deconvolution of peaks, peak heights were used into a relative quantitation between Brønsted and Lewis acid sites. From this approach, one can conclude that after Pd loading there has been a decrease in Pd/HBEA Bronsted acid site at 1542 cm^{-1} (H_B/H_L ratio = 0.29/1.00) when compared to HBEA Bronsted acid site at 1542 cm^{-1} from (H_B/H_L ratio = 0.64/1.00).

Evaluation of n-hexane conversion isolated and mixed with DX over reaction time

A scheme illustrating the difference of pure n-hexane conversion and mixed with DX are depicted on Supplementary Figure 3.

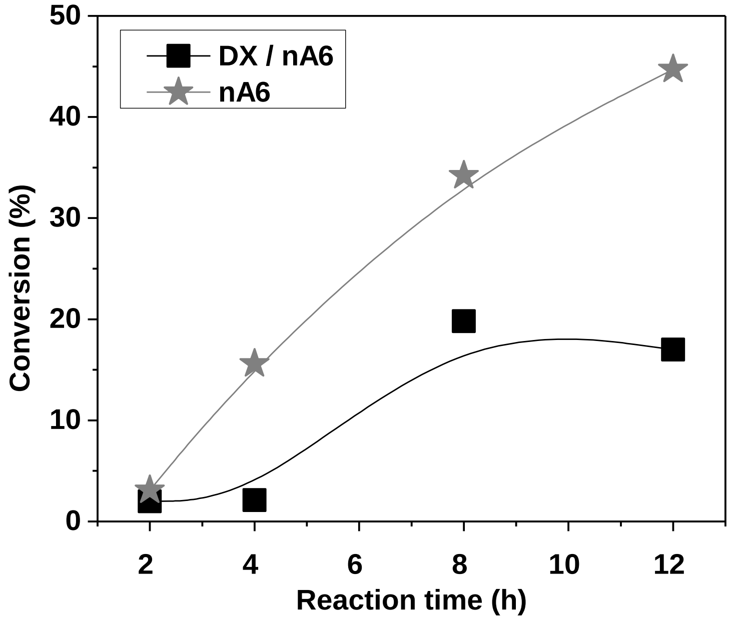

Supplementary Figure 3 | N-hexane conversion of . Conditions: 250°C; 30 bar H_2; 90g n-hexane; 5g DX ;10g Cat; Catalyst/DX = 2/1

Differences on n-hexane conversions profiles suggests a competition for acid sites between n-hexane and DX at the first 4h and a n-hexane and coke/hydrocarbons from 8 to 12h of reaction. When isolated, n-hexane conversion presents an ongoing increase over reaction time.

Product distributions of A_{7+} compounds over different reaction times

The composition of A_{7+}, produced from experiments carried out at different reactions times, is shown in more detail on Figure S2.

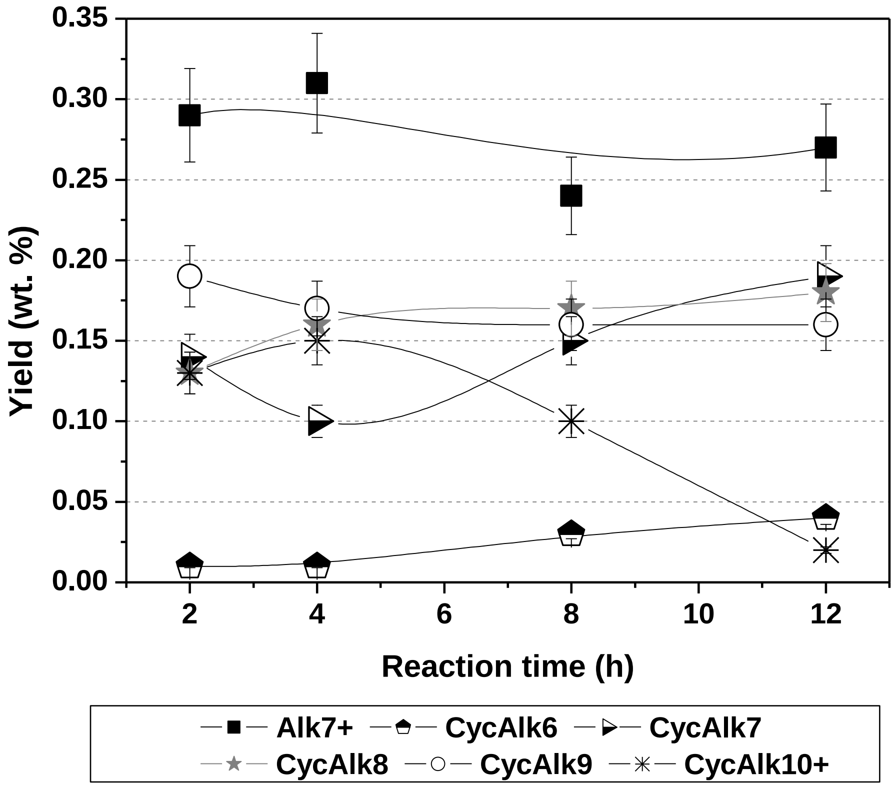

Supplementary Figure 4 | Product distribution of alkanes with 7 or more carbons including methyl cyclohexane and cyclohexane. Conditions: 250°C; 30 bar H_2; 90g n-hexane; 5g DX ;10g Cat; Catalyst/DX = 2/1

Data on Figure S2 shows that Alk_{7+} selectivity remained almost unaltered during reaction. Cycloalkanes yields split in two trends: $CyAlk_6$, $CyAlk_7$ and $CyAlk_8$ yields increased with increase of time reaction whereas $CyAlk_9$ and $CyAlk_{10+}$ yields decreased with increase in reaction time. From results on Figure S2 it is clear that $CyAlk_{10+}$ compounds (and at less extent $CyAlk_9$ compounds) start to undergo hydrogenolysis from 4h of reaction. Thus far, results showed on Figure S2 endorses that the increase in reaction time favored the decrease in 'heavier' products yield (i.e CyAlk9 an CyAlk10+ compounds).

Coke analysis

On Supplementary Figures 5A and 5B are illustrated thermogravimetric curves obtained from n-hexane and DX/n-hexane hydrotreatment using Pd/HBEA catalyst.

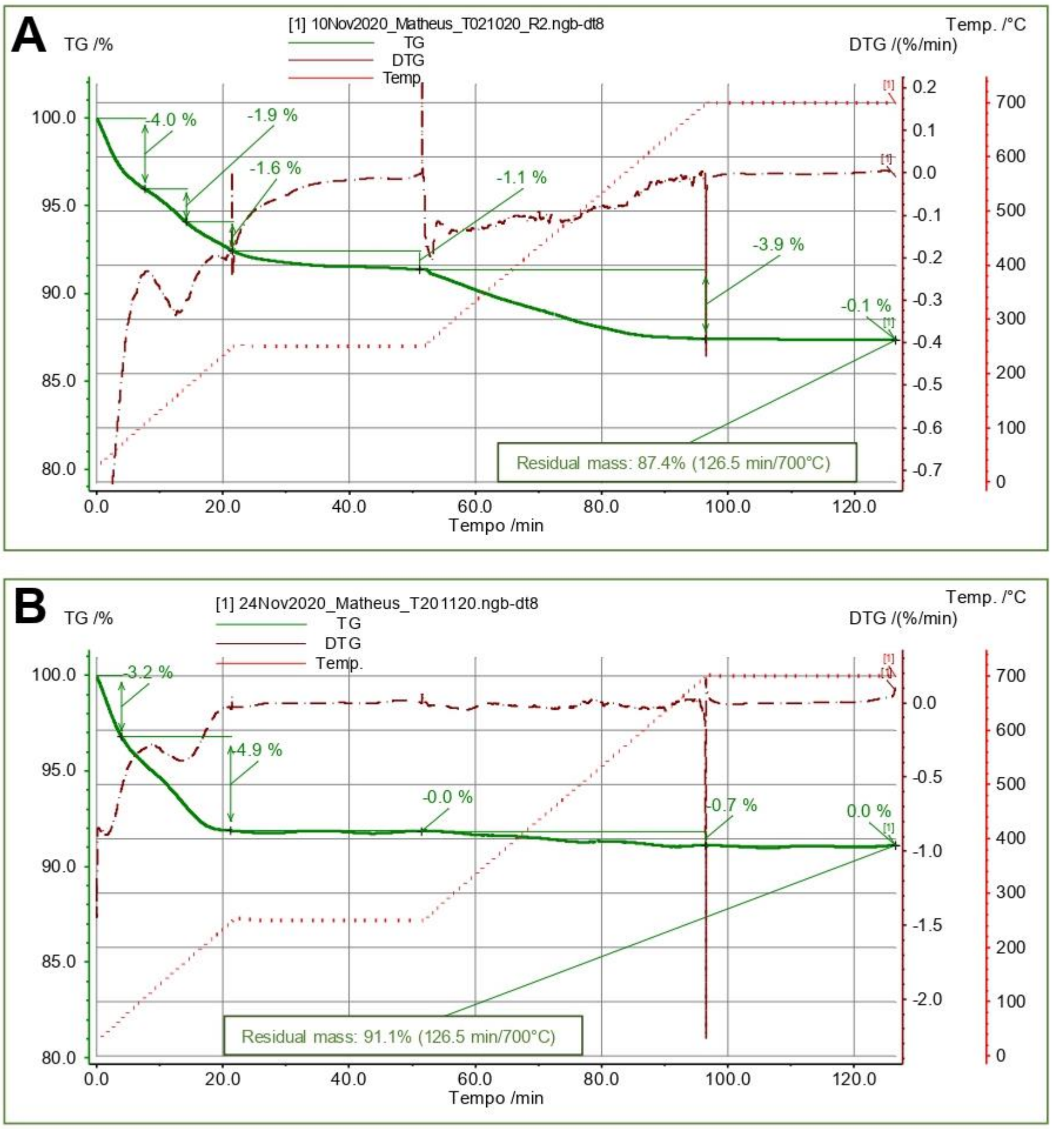

Supplementary Figure 5 | Thermogravimetric curves obtained from coke analysis on Pd/HBEA spent catalysts yielded through DX/n-hexane (A) and pristine n-hexane (B) hydrotreatment reactions. General reaction conditions: 250°C; 30 bar H_2; 4h; 90g n-hexane; Catalyst/DX ratio (wt.): 2/1 = 10g Cat / 5g DX.
